# Construction and Characterization of Phthalocyanine-Loaded Particles of Curdlan and Their Photosensitivity

**DOI:** 10.3390/ijms19113323

**Published:** 2018-10-25

**Authors:** Zonglin Liu, Dongfeng Wang, Xun Sun, Qingjie Sun, Yanjiang Wu, Ying Xu

**Affiliations:** 1 College of Food Science and Engineering, Ocean University of China, Qingdao 266003, China; linsuga420@gmail.com (Z.L.); wangdf@ouc.edu.cn (D.W.); sunxun137@sina.com (X.S.); 2 College of Food Science and Engineering, Qingdao Agricultural University, Qingdao 266109, China; phdsun@163.com; 3 Dalian Bangchuidao Marine Products Co., Ltd. Dalian 116000, China; sunxw0809@gmail.com

**Keywords:** curdlan, phthalocyanine, supramolecular, assemblies

## Abstract

To optimize the physicochemical properties of phthalocyanine (PC), we examined its behavior in particles of triple helix glucan curdlan (CUR). CUR was denatured and renatured in DMSO, in the presence of PC. Infrared spectroscopy and transmission electron microscopy (TEM) showed that PC and CUR formed an inclusion complex, in which PC was trapped inside CUR molecules. This redshifted the absorption peak of PC, which would improve its usefulness as a photosensitizer, because infrared light can penetrate more deeply into human tissues. The conductivity of the solution of CUR-PC was higher than the conductivities of either a CUR solution or a PC dispersion, indicating that CUR-PC is more water soluble than PC. In addition, CUR-PC was highly stable in water. Thus, the use of CUR as a carrier of PC improves several of its physical properties. PC is used as a photosensitizer for killing cancer cells, but its use is hampered by its low solubility. Further, its absorption range limits its use to a depth of 1–3 mm in tissues. CUR-PC, with its high solubility and infrared absorption peak, was highly effective as a photosensitizer. It killed 84% of HeLa cells under 15 min of long wavelength radiation and had little cytotoxicity in the absence of light. These results demonstrate that CUR-PC has promise as a photosensitizer, as well as provide theoretical support for a wide range of applications for PC and CUR.

## 1. Introduction

**Figure 1:**
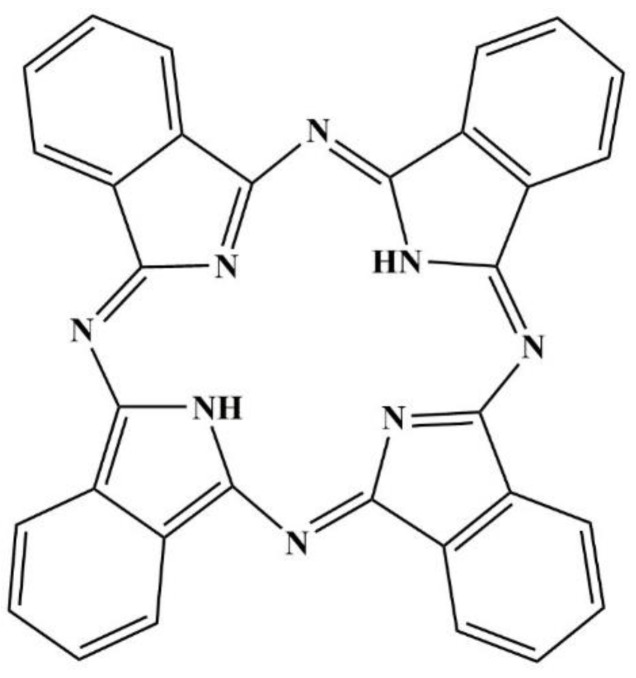
The molecular structure of phthalocyanine.

Phthalocyanine (PC) and its derivatives are a class of synthetic compounds that are closely linked to natural porphyrins. They have many uses as pigments and dyes [1,2]. PC has a planar and symmetric structure, with a high level of conjugation, as shown in Figure 1, which gives it a stable and intense blue color. PC derivatives can also improve the performance of semiconductors [3]. Based on these features, PC is extensively used in the fields of medicine, photonics, electronics, and energy conversion [4,5].

Presently, PC is attracting a lot of attention, because it has potential use in treating cancer. In the early 20th century, PC was found to have an affinity for neoplastic tissues, allowing it to be selectively transported, retained, and enriched in tumor tissues. It also has low cytotoxicity [6]. These properties, in addition to its optical absorption properties, allow PC and its derivatives to act as photosensitizers that can efficiently kill tumor cell tissues, without affecting the surrounding healthy tissues [7,8,9,10]. Traditional photosensitizers have an excitation wavelength of about 400–600 nm. Because human chromophores absorb light in this range, traditional photosensitizers are useful only within 1–3 mm of the surface of a tissue [11]. This means that, for a photosensitizer to be effective, it needs to have an absorption band over 700 nm. PC has a strong absorption in the ultraviolet and visible portions of the light spectrum [12], as well as favorable chemical and thermal stability [13,14]. Another desirable property is water solubility. However, PC has an absorption peak at 670 nm and low water solubility, which detracts from its otherwise excellent properties [15]. One approach to solving these problems is to synthesize new PC derivatives with improved solubility and optical properties [7,16]. However, synthesis of photosensitizers is time-consuming and expensive. Here, we tried a different approach, in which we attempted to improve the biological properties of PC by combining it with a biomacromolecule—such as a polysaccharide—to form a supramolecular assembly.

Triple helix (1→3)-β-d-glucans are widely distributed in organisms, such as bacteria, yeasts, fungi, and plants [17]. The individual chains of these glucans—such as curdlan, lentinan, and schizophyllan—are helical in solution and, under natural conditions, spontaneously form right-handed triple helices [18,19,20,21,22,23]. In addition to their β-(1→3)-d-glycosyl linkages, they also have some (1→6)-d-glycosyl side-chains [24]. When (1→3)-β-d-glucans form triple helices, they tend to interact with many other guest molecules. This gives them some unique biological properties, such as high immune activity, anti-HIV and antitumor activities, and other medically useful properties [25,26,27].

Curdlan (CUR)—the first identified, simplest, and best known (1→3)-β-d-glucan—is produced by *Agrobacterium biovar* [28]. It has been widely used in the food industry as a gelling, bulking, and coating agent, because of its edibility (although, it is not digested) and can be produced in large amounts, with high quality [29]. Triple helix curdlan (t-CUR) can be unwound into single chains (s-CUR) by being dissolved in dimethyl sulfoxide (DMSO) and can be renatured into the original triple helix structure by replacing DMSO with water [30]. T-CUR can also be unwound into s-CUR in strong alkaline solution and renatured, when the solution is neutralized [31]. Schizophyllan can form complexes with some polynucleotides [32,33], as well as a one-dimensional composite of permethyldecasilane [34], and can interact with small molecular dyes—such as phenylazobenzene and pyrrole derivatives—to form nanomolecular particles, with different optical properties [35,36].

These discoveries encouraged us to investigate the use of triple helix glucans as carrier molecules. Here, we describe the construction of a curdlan and PC complex (CUR-PC). We expected that t-CUR could be dissociated into s-CUR and renatured, which would make it useful for constructing PC-loaded particles. Our goal was to create stable CUR-PC complexes with good solubility and electro-optical activity, as well as to validate their use in suppressing cancer cells. Because our method was simple and inexpensive, we believe that it will be useful for developing a variety of photosensitizers.

## 2. Results

### 2.1. Preparation and Spectral Analysis of CUR-PC

We prepared 1 molar solutions of CUR and PC in DMSO. The DMSO caused the triple helix of CUR to unwind. The two solutions were mixed, at different volume ratios. The combined solution was then diluted with nine volumes of water, which induced the CUR to reform into a triple helix, entrapping PC molecules in the process. The mixture was allowed to stand for 15 min at room temperature, during which time CUR-PC particles formed and precipitated. See Section 4.2 for the detailed method.

**Figure 2:**
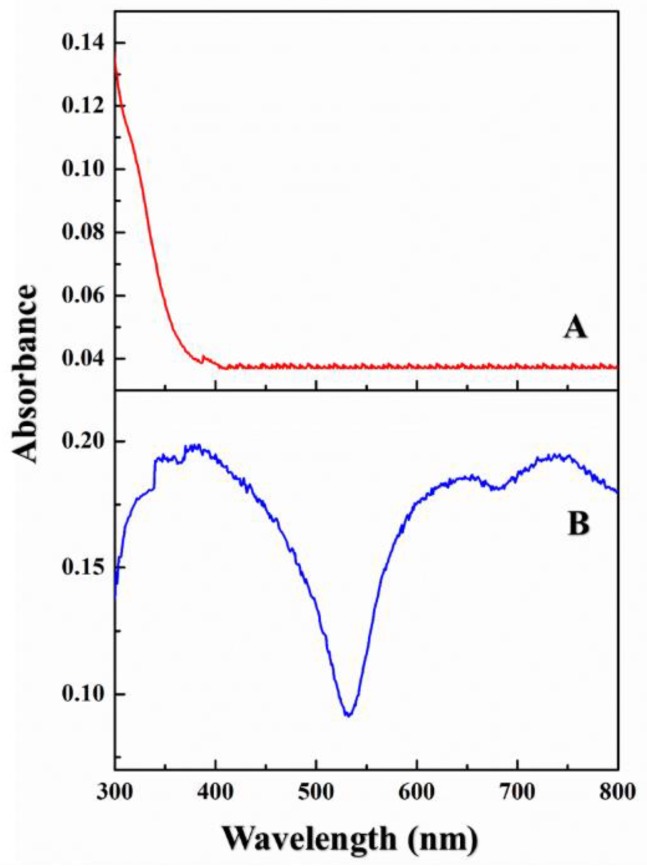
UV-vis spectra of CUR in water (**A**) and CUR-PC in water (**B**).

**Figure 3:**
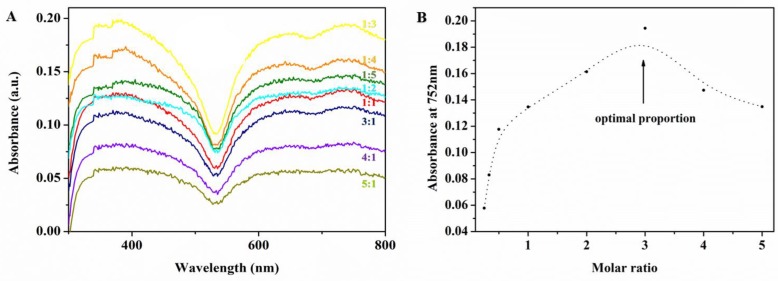
(**A**) UV-vis spectra of different volume ratios (CUR/PC); (**B**) absorbance at 752 nm of different volume ratios (CUR/PC).

CUR by itself absorbed in the UV range only, as shown in Figure 2A. PC is not water soluble, but electron spectroscopy showed that it had two absorption bands: The B band (typically between 250 and 350 nm) and the Q band (typically between 600 and 700 nm; data not shown) [10,37]. The electron spectra of PC derivatives were similar. The B and Q absorption bands were caused by π-electron transitions on the PC ligand ring. In the composite CUR-PC—which was water-soluble, as shown in Figure 2B—the B and Q bands were broadened. CUR-PC also had a new absorption peak at 752 nm, the result of a redshift in the Q band. Figure 3A shows the absorption spectra of different CUR:PC molar ratios, starting from a weak 1:5 ratio to a strong 5:1 ratio. As the molar ratio increased, absorption in the B and Q bands first increased and then, at a molar ratio of 3:1, began to decrease. This is also shown by the absorbance at 752 nm, which peaked at a molar ratio of 3:1, as shown in Figure 3B.

### 2.2. Particle Observation and Effective Particle Size Analysis

**Figure 4:**
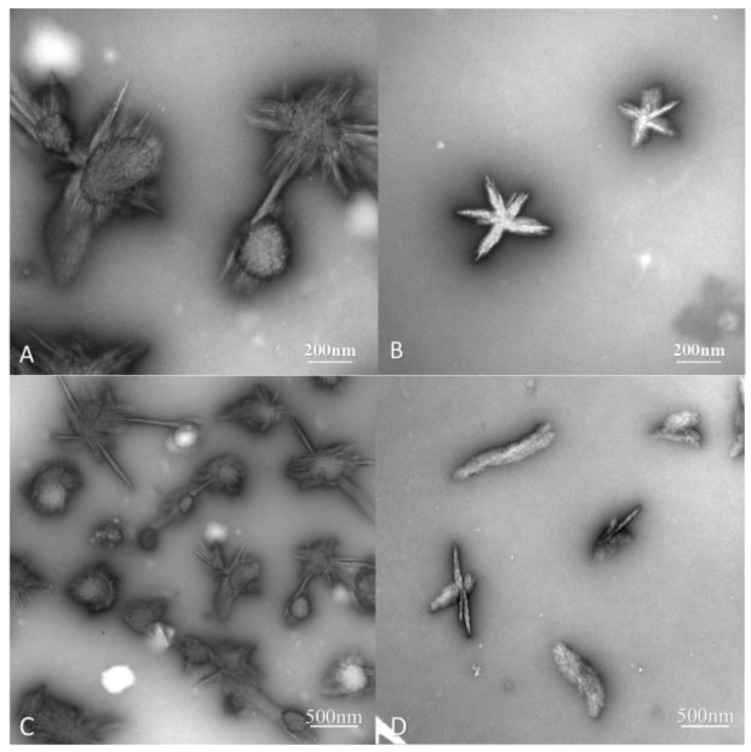
Transmission electron microscope (TEM) image of CUR-PC (**A**,**C**) and CUR (**B**,**D**).

**Figure 5:**
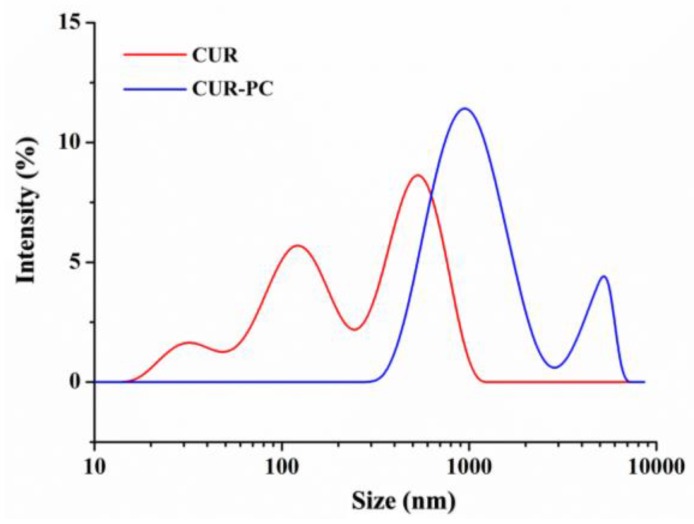
Particle size of renatured CUR and CUR-PC.

Under the transmission electron microscope (TEM), CUR particles appeared irregular and star-shaped, with a size of about 500 nm—as shown by Figure 4B,D—while CUR-PC particles at a CUR:PC molar ratio of 3:1 were larger and more irregular, as shown by Figure 4A,C. The average particle sizes of CUR-PC and CUR, as measured by ImageJ, were 760 ± 260 nm and 460 ± 200 nm (mean ± SD), respectively. Particle sizes were also measured independently, with a Zeta-sizer Nano ZS90. CUR had 3 peaks around 30, 150, and 800 nm, while CUR-PC was more homogeneous, with a main peak at about 970 nm and a secondary peak at 5500 nm, as shown in Figure 5.

### 2.3. Fourier Transform Infrared (FTIR) Spectroscopy

**Figure 6:**
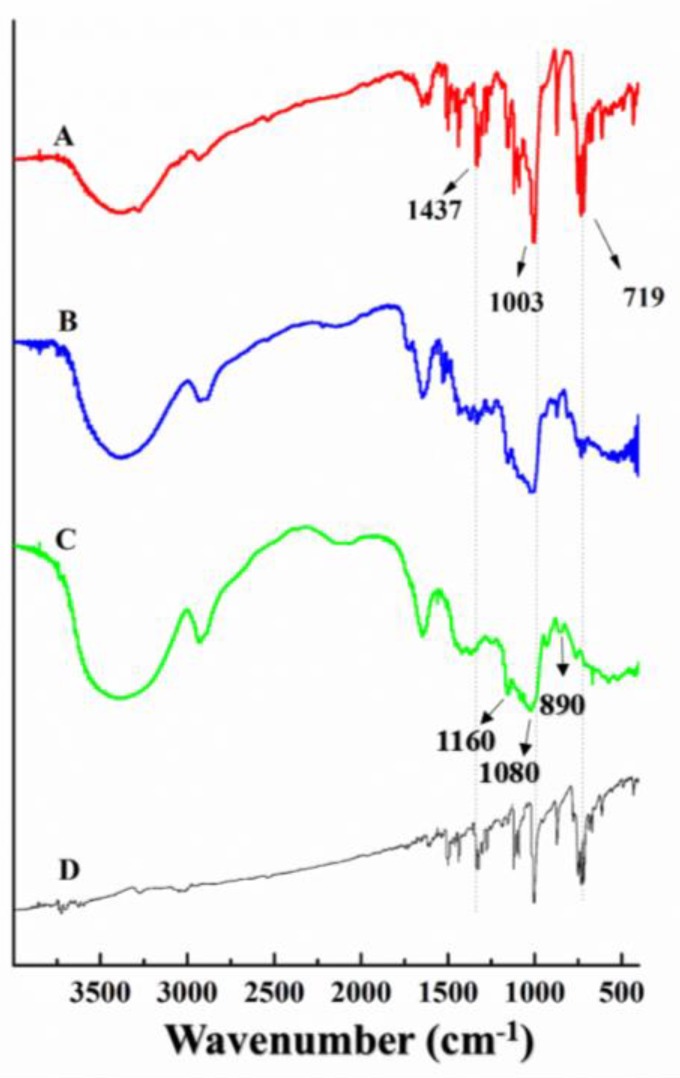
FTIR spectra of CUR mixed with PC (physical mixture) (**A**), CUR-PC (**B**), CUR (**C**), and PC (**D**).

The loading of PC into CUR was examined by FTIR. The characteristic infrared absorption spectrum of CUR had peaks corresponding to β-(1,3) glucan linkages at 890, 1080, and 1160 cm^−1^, as shown in Figure 6, spectrum C. The PC spectrum (spectrum D) had characteristic peaks at 719, 1003, and 1437 cm^−1^, which were attributed to the C–H, out-of-plane bending vibration peak. Peaks at 1568 and 1584 cm^−1^ were attributed to benzene ring C–C stretching. A peak at 1428 cm^−1^ was attributed to benzene ring stretching, as well as C–H and N–H in-plane bending. The carbon-nitrogen-conjugated double bond was considered a functional group of PC. The spectrum of a physical mixture of PC and CUR (i.e., not the CUR-PC complex) had characteristic PC peaks at 719, 1003, and 1437 cm^−1^ (spectrum A), along with other peaks related to CUR (spectrum C). However, these peaks were not observed in the CUR-PC spectrum (spectrum B), which suggested that the PC was encapsulated by nanoparticles. Moreover, the peaks presented in the physical mixture were not present in the CUR-PC spectrum, which showed that the nanoparticles formed through an interaction between PC and CUR.

### 2.4. Conductivity

**Figure 7:**
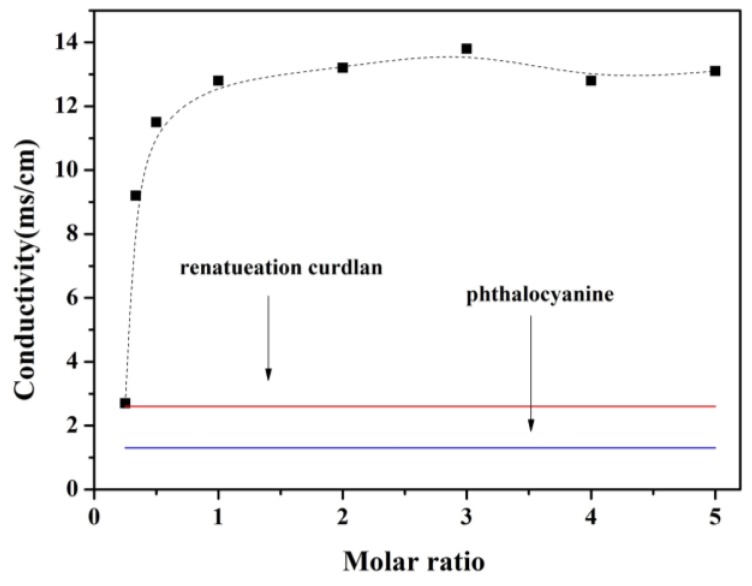
Conductivity of the samples.

At low volume ratios (≤1), the conductivity of CUR-PC solutions increased dramatically with increasing volume ratio, reaching a plateau at a volume ratio of 1, as shown in Figure 7. For comparison, ultrasonically dispersed PC in water (1 mg/mL) had a conductivity of 1.3 μSiemens/cm (μS/cm)—as shown by the blue line in Figure 7—and the conductivity of renatured CUR was 2.6 μS/cm, the red line in Figure 7. Thus, the CUR-PC complex had a much higher conductivity than its individual components.

### 2.5. Stability of the CUR-PC Solution

**Figure 8:**
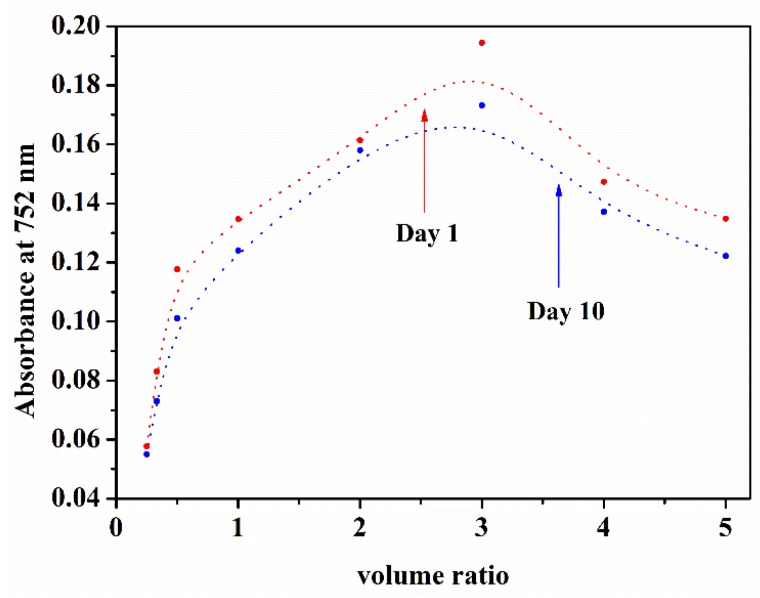
Absorbance at 752 nm of different volume ratios (CUR/PC).

To determine the stability of the CUR-PC solutions, solutions with different molar ratios were allowed to stand in the dark, at room temperature. After 10 days, the solutions were vortexed again, to break up aggregates. The quantity of CUR-PC was determined by measuring its absorbance at 752 nm. After ten days, the CUR-PC content decreased below 10%, at each molar ratio, as shown in Figure 8. These results showed that PC embedded in CUR was highly stable.

### 2.6. Inhibition of Cancer Cells by CUR-PC

**Figure 9:**
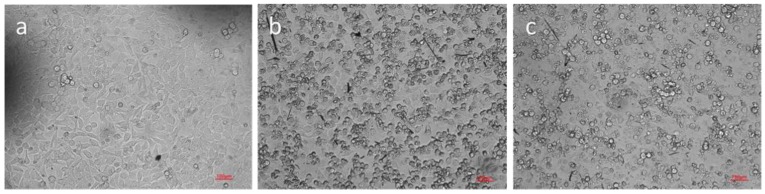
Microscopy images of HeLa cells: Control group (**a**), cultured with CUR-PC (**b**), and cultured with CUR-PC, then exposed to long-wavelength light irradiation (**c**). Scale bar = 100 μm.

**Figure 10:**
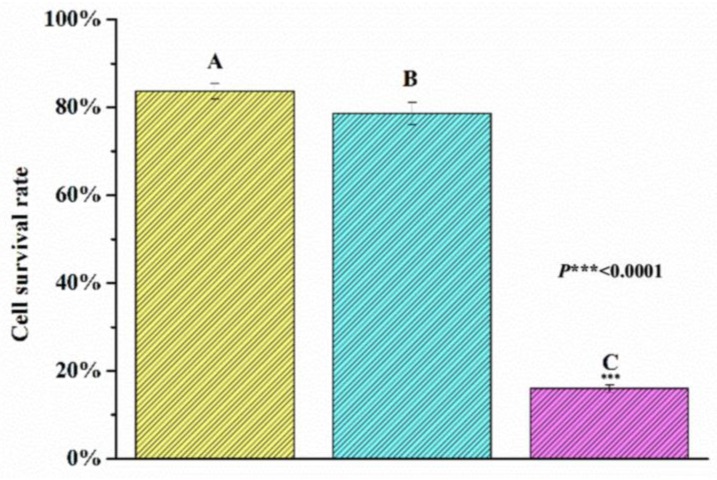
Cell survival rate of the HeLa cells treated with long-wavelength light irradiation: Control group (**A**), cultured with CUR-PC (**B**), and cultured with CUR-PC, then exposed to long-wavelength light irradiation (**C**).

Healthy HeLa cells had elongated shapes and were attached to a culture plate, as shown by Figure 9A. When the cells were cultured with 0.25 mg of CUR-PC/mL culture medium, in the dark, for 15 min, some of the cells took on a spherical shape, indicating that they were probably dead, as shown by Figure 9B. These results indicated that CUR-PC had good biocompatibilities that did not generate a cytotoxicity effect to the cells. To test the ability of CUR-PC in acting as a photosensitizer, HeLa cells were treated with CUR-PC, at a final concentration of 0.25 mg/mL, for 2 h, and then illuminated with a long wavelength (>750 nm) light, for 15 min. Almost all the cells treated in this way died, as shown by Figure 9C. Counting of dead cells with trypan blue revealed that most (84%) cells had survived exposure to long-wavelength light by itself—as shown by Bar A, Figure 10—and that 82% of the cells had survived exposure to CUR-PC by itself, as shown by Bar B, Figure 10. In contrast, only 16% of the cells survived treatment with both long wavelength light and CUR-PC, as shown by Bar C, Figure 10. Thus, CUR-PC dramatically increased the cell-killing effect of long-wavelength light.

## 3. Discussion

Through denaturation and renaturation, helical polysaccharide chains—such as a single helix of v-amylose and a triple helix of Schizophyllan—could form functionalized nanostructures, by incorporating multiple hydrophobic guest molecules into their hydrophobic cavities [33,38,39,40]. Two nanostructures of this type, loaded with PC, have been developed for drug delivery [41,42]. Using a polymer as a carrier, PC has broad application prospects in drug delivery. Our nanoparticles, made of triple helix CUR loaded with PC, have sizes of about 970 nm, which is much larger than CUR particles. This suggests an interaction between the two components. This was confirmed by the infrared spectra. In addition, the morphology of the CUR-PC complex was completely different from the morphology of renatured CUR, as shown by Figure 4, which suggested that PC was trapped in the CUR cavity. This could cause changes in the complex’s physical properties, including an increase in conductivity, as shown in Figure 7. Through interactions of this type, supramolecular structures can be created.

In this study, different molar ratios of CUR and PC were mixed in DMSO and diluted with water, to make CUR-PC. The UV-vis spectra of all solutions could be measured, and each had a redshifted absorption maximum (752 nm). Single-molecule-dispersed PC molecules have strong absorption, around 690 nm [43], corresponding to the π–π* transition of the phthalocyanine conjugated ring π electron, from the highest occupied orbit to the lowest occupied orbit. The redshift from 690 nm to 752 nm may have been related to the presence of J-type aggregates in phthalocyanine [44,45]. This would be beneficial for practical applications of CUR-PC. For example, in photodynamic therapy (referred to as PDT), ideal photosensitizer should have strong absorption in the wavelength range of 600–800 nm. Practical applications of PC complexes often involve the Q band of their absorption spectra. CUR-PC absorbed in this range and was very stable, as shown by Figure 8. Together, these properties could help to explain CUR-PC’s ability in acting as a photosensitizer and killing cancer cells.

## 4. Materials and Methods

### 4.1. Materials

Curdlan was obtained from Shanghai Yuanye Biological Technology Co., LTD (Shanghai, China). Phthalocyanine was acquired from Tokyo chemical industry CO., LTD (Tokyo, Japan). DMSO was obtained from Sigma Chemical Company (St. Louis, MO, USA).

### 4.2. Creation of CUR-PC by DMSO Treatment and Effective Particle Size Analysis of Complex

Curdlan was solubilized with DMSO, as previously described, with some modifications [35]. The molecular weight of the repeating unit of curdlan was 486, and the molecular weight of phthalocyanine was 514. We then prepared 1 M of each solution with DMSO and mixed each solution with its corresponding volume ratio (CUR/PC = 3, 2, or 1). Because of the low solubility of the PC in DMSO, the mixture was subjected to several seconds of ultrasonication, in an ultrasonic cleaner. Different volumes of PC solution and CUR solution were mixed to obtain a given volume ratio. Then, 4 volumes of water were added, while stirring under a magnetic stirrer, for 15 min. The final composition of water/DMSO (*v*/*v*) was 90/10. After running through these steps, the s-CUR interacted with PC, in the DMSO. During this process, release of heat could be felt. Different volume ratios of CUR/PC solution were prepared, to confirm the optimal proportion of PC and CUR. The self-assembling of PC proceeded, and the CUR-PC could be obtained. UV-vis spectra were obtained, with a 2102PC spectrophotometer (Unico Instruments Co., Ltd., Shanghai, China), to detect the presence of PC and CUR complexes. The solution was dialyzed against 500 ml of water, with a 12,000 MWCO dialysis tubing bag, for 48 h, and then frozen and lyophilized. Renatured CUR was prepared in the same manner, without PC addition.

### 4.3. CUR-PC Particle Size

Freeze-dried, renatured CUR and freeze-dried CUR-PC were weighed and separately dissolved in water, at a concentration of 1 mg/mL. The effective particle size was determined by dynamic light scattering technology (DLS), using a Zeta-sizer Nano ZS90 (Malvern Instruments Ltd., Malvern, UK), at 25 °C.

### 4.4. Infrared Spectroscopy

The samples were embedded in KBr (potassium bromide). IR spectra were obtained with an FTIR spectrometer (NicoletiS10, Thermo Scientific, Rochester, NY, USA). For both samples, 64 interferograms, with a resolution of 4 cm^−1^ were co-added, and the spectral information obtained, over a frequency window of 4000 to 400 cm^−1^.

### 4.5. Transmission Electron Microscopy

A droplet of the solution was put onto a carbon support film, on a copper grid. Excess was removed after 5–10 min, with filter paper. A droplet of 1% (*w*/*v*) phosphotungstic acid was put onto the grid and removed with a filter paper, after 5–10 min. After drying at room temperature, the grid was observed, using a TEM device (JEM-1200EX, JEOL Ltd., Tokyo, Japan), at a voltage of 100 kV.

### 4.6. Conductivity Measurements

A DDS307 conductivity meter was used to determine the conductivities of CUR-PC, made by different volume ratios, ultrasonic-treated dispersion of PC, and CUR in water.

### 4.7. Stability

CUR-PC solutions were left at room temperature for 10 days. The content of these CUR-PCs was measured by UV-vis. UV-vis absorption measurements were performed, using a 2102PC spectrophotometer (Unico Instruments Co., Ltd.), equipped with 1.0 cm quartz cells.

### 4.8. HeLa Cell Survival

HeLa cells were cultured in 96-well plates, at 5 × 10^4^ cells per well, in a DMEM (Dulbecco’s Modified Eagle Medium) medium, containing 10% fetal bovine serum. The cells were treated with the CUR-PC solution (0.25 mg/mL, 100 μL) in a DMEM medium. After 2 h of incubation, HeLa cells were treated with or without light treatment (light power density: 150 mW/cm^2^) for 15 min. HeLa cells with only the light treatment were used as a control group. After irradiation, all plates were placed in a humidified incubator for 2 h. The metamorphoses of the cells were observed with an inverted microscope. The cells were stained with trypan blue and dead cells were counted with a cell counter (Beckman Coulter, Fullerton, CA, USA).

## 5. Conclusions

In conclusion, we synthesized a phthalocyanine-loaded particle, based on glucan curdlan (CUR-PC), via DMSO treatments. PC and CUR interacted to form an inclusion complex, which improved conductivity and excellent stability. Exposure of HeLa cells to CUR-PC and long-wavelength light killed most cells. The PC-loaded nanoparticles developed in this study may serve as effective delivery systems for photosensitizers and other pharmaceutical products.

## Abbreviations

CUR	curdlan
CUR-PC	PC and CUR complex
PC	phthalocyanine
